# Medical Legislation

**Published:** 1845-12-01

**Authors:** 


*Medical Legislation.*—On this subject, the Editor of the New-York Journal of Medicine, remarks as follows in an article advocating the proposed National Convention. "In our opinion it belongs legitimately to the general government to regulate the qualifications of those who undertake the practice of the healing art in the United States, and we find the authority conferred in that clause of the Constitution, which confers on it the power of "*providing for the general welfare*"—for if the practice of medicine is not connected with the "general welfare," we know not what is. Indeed, the government has already taken this power into its own hands in granting patents for certain modes of practice, as *Thomsonism*, and a great variety of quack pills, mixtures, and other secret preparations; we now go for an extension of its jurisdiction, and thus providing some general scheme for the education and licensing of medical men similar to that now in contemplation by the parliament of Great Britain. In no other way, in our judgment, can the desired reforms be brought about. In no other way can an uniform system be carried into operation throughout the entire Union." No. for Nov. 1845.

